# Evaluation of clinical significance of decompressive suboccipital craniectomy on the prognosis of cerebellar infarction

**DOI:** 10.20407/fmj.2018-010

**Published:** 2018-12-06

**Authors:** Yoshio Suyama, Shinichi Wakabayashi, Hiroshi Aihara, Yusuke Ebiko, Hiroshi Kajikawa, Ichiro Nakahara

**Affiliations:** 1 Department of Neurosurgery, Suiseikai Kajikawa Hospital, Hiroshima, Hiroshima, Japan; 2 Department of Comprehensive Strokology, Fujita Health University School of Medicine, Toyoake, Aichi, Japan

**Keywords:** Cerebellar infarction, Decompressive suboccipital craniectomy, Ventricular drainage, Outcome

## Abstract

**Objective:**

The decision of whether and/or when to treat cerebellar infarction surgically remains controversial. We investigated the effectiveness of decompressive suboccipital craniectomy (DSC) for treating cerebellar infarction and the prognostic factors that affect the surgical results.

**Methods:**

From October 2006 to June 2017, a total of 14 consecutive patients (12 men, 2 women; mean±SD age 65±12 years, range 42–84 years) were admitted to our hospital and underwent DSC at the time of admission or during their hospitalization. Inclusion criteria were (1) a level of consciousness below Glasgow Coma Scale (GCS) 13, and/or (2) brainstem compression and/or obstructive hydrocephalus caused by brain edema due to cerebellar infarction. Ventricular drainage was performed simultaneously or later, according to the surgeon’s decision.

**Results:**

At the 90-day point, 12 of the 14 patients (85.7%) had survived, 10 (71.4%) of whom were independent (modified Rankin scale ≤2). Four (28.6%) were either completely dependent or dead. Comparisons between good and poor prognoses showed that the factors affecting the prognosis were lesions other than the cerebellar infarction (*p*<0.01) and/or obstructive hydrocephalus (*p*<0.05).

**Conclusions:**

Early DSC should be considered for treating cerebellar infarction in patients with GCS 13 or worse. A poor prognosis is inevitable in patients whose infarction is combined with other location than the cerebellum but in those who already have obstructive hydrocephalus at the time of surgery.

## Introduction

Cerebellar infarction and associated brain edema due to brainstem compression or obstructive hydrocephalus causes consciousness disturbance. In such cases, the mortality rate is reported to be 84%^1^ when decompressive suboccipital craniectomy (DSC) is not performed. Criteria for patient selection and the timing of the operation are not yet established, although there are several reports that DSC is effective.^1–3^ We studied 14 patients who underwent DSC for cerebellar infarction and reviewed the literature on the indications for, and timing of, surgical intervention, as well as the factors related to the postoperative prognosis.

## Methods

We retrospectively analyzed the medical records of 14 consecutive patients who underwent DSC for cerebellar infarction between October 2006 and June 2017 (10 years 9 months) at the institution (SKH) of the first author (YS). Emergent surgery was indicated if one or both of the following criteria were observed at admission or after hospitalization: (1) level of consciousness below a Glasgow Coma Scale score of 13; and (2) brainstem compression and/or obstructive hydrocephalus caused by brain edema due to the cerebellar infarction. For patients who met the criteria, we evaluated their general condition to determine if there was any obstacle to general anesthesia or being in the prone position for approximately 4 h. Then, after acquiring informed consent from patient’s family, we performed emergency surgery.

A skin incision was made in a median longitudinal fashion from 2–3 cm above the inion to the fifth cervical vertebral process level, with an additional T-shaped transverse incision measuring approximately 8 cm at its upper end. In each case, suboccipital craniectomy was performed until the foramen was opened. If intraoperative findings indicated that decompression achieved by the suboccipital craniectomy was insufficient, laminectomy of the first cervical vertebra was performed. An inverted Y-shaped incision was made on the dura mater, and internal decompression (e.g., by removing infarcted brain tissue) was performed in each case. In addition to the external decompression, artificial dura mater composed of GORE-TEX membrane was used for dural plasty. Ventricular drainage was performed when hydrocephalus was observed preoperatively or if it was anticipated to occur even after DSC, based on the intraoperative findings.

From each patient’s medical records, we retrospectively determined and recorded the patient’s age, sex, time from onset until surgery, infarction at other locations, pathology of the cerebellar infarction, hemorrhagic infarction, hydrocephalus, and the infarction volume. Statistically significant differences between these factors regarding the prognosis were examined. The volume of the infarcted lesion was measured on an magnetic resonance imaging (MRI) diffusion-weighted image using AZE virtual place Fujin Raijin 360 (AZE, Tokyo, Japan). The prognosis was evaluated 90 days after onset and was expressed according to the modified Rankin Scale (mRS). This study was reviewed and approved by the institutional ethics committee of the institution (SKH) of the first author (YS).

## Results

Table 1 shows the details of the 14 patients studied (12 men, 2 women; mean±SD age 65±12 years, range 42–84 years). Cerebellar infarction was caused by cardiogenic cerebral embolism in 9 patients, atherothrombotic cerebral infarction in 4, and artery-to-artery embolic infarction by undetermined cause in 1. Hemorrhagic infarction was found in 9 patients. The posterior inferior cerebellar artery, superior cerebellar artery, and anterior inferior cerebellar artery were involved in 12, 5, and 1 patient, respectively. There was multiple involvement among these three dominant arteries perfusing the cerebellum in 7 patients. Three of them had infarction in the posterior cerebral artery (n=2) or middle cerebral artery (MCA) (n=1) territories. As a result, brainstem compression and/or hydrocephalus was observed on computed tomography (CT) or MRI in all cases before DSC.

The mean±SD time (hours:minutes) from onset to surgery was 60:30±44:21 (range 16:00–157:10). The mean±SD volume of the infarcted lesion immediately before DSC was 64.3±19.2 ml^3^ (range 33.4–104.7 ml^3^). There were no complications associated with the surgical procedure. After 90 days, the mRS was ≤2 in the 10 patients (71.4%) who did not require total assistance and ≥3 in the two patients (14.3%) who required total assistance. The other two patients had died. Severe cerebral infarction of the occipital lobe was the cause of the poor prognosis in two patients (cases 7 and 12). In addition, two patients died, one from severe heart failure (case 5) and one from brainstem hemorrhage (case 8).

Comparing the good- and poor-prognosis groups showed no significant difference in age, sex, time from onset to operation, or the pathology of the cerebellar infarction, hemorrhagic infarction, or myocardial infarction. Significant differences were found in “Infarction in areas other than the cerebellum” and “obstructive hydrocephalus” among the evaluated factors (Table 2).

## Discussion

There is still no consensus of whether (or when) to use a surgical operation in cases of cerebellar infarction accompanied by cerebellar edema and consciousness disorder.^4^ The frequency of cerebellar infarction with cerebellar edema and symptoms is reported to be 17%–54%.^4,5^ In addition to cytotoxic edema caused by the cerebral infarction itself, the mechanism of this condition includes vasogenic edema due to the natural recanalization of occluded vessels,^6^ as well as hemorrhagic changes, direct compression of the brainstem with upward herniation of the superior vermis cerebelli through the tentorial notch, downward herniation of the cerebellar tonsils through the foramen magnum, and obstructive hydrocephalus, resulting in disturbed consciousness.^4,5^ As already suggested, surgical treatment for such patients remains controversial.

On one hand, the American Heart Association/American Stroke Association (AHA/ASA) guideline^3^ states that surgical treatment for cerebellar infarction is as follows for class I, Level of Evidence B, “Suboccipital craniectomy with dural expansion should be performed in patients with cerebellar infarctions who deteriorate neurologically despite maximal medical therapy.” The Guidelines for Stroke Treatment in Japan 2015^7^ offer a definitive recommendation to perform DSC in cases of cerebral infarction of the unilateral cerebral hemisphere caused by MCA occlusion (grade A). There is insufficient evidence, however, to support surgical treatment for cerebellar infarction (grade C1), although several reports have found that decompression surgery is effective for cerebellar infarction.^1–3^

Feely^1^ reported on 55 patients with cerebellar infarction who became comatose. The mortality rate was 84% when DSC was not performed and 28% when it was performed. Thus, DSC was reported to be effective.^1^ Ogasawara et al.^3^ reported on 10 patients with cerebellar infarction whose consciousness had deteriorated. Following DSC, 7 of the 10 patients had a good recovery. Tsitsopoulos et al.^2^ examined 32 patients who underwent DSC, 17 (53.1%) of whom had a good prognosis.

A poor preoperative state of consciousness is reported to be related to a poor prognosis. In such cases, DSC has improved the prognosis in half of comatose patients and in patients without brainstem infarction^2,3,8^. Regarding age, the DENSITY study, which examined broad cerebral infarction in the MCA region, showed surgical effectiveness in patients <70 years of age.^9^ However, in patients with cerebellar infarction, Tsitsopoulos et al.^10^ reported that age did not affect prognosis even if patients were >70 years. Regarding the patient’s state of consciousness when deciding on surgery, Ogasawara et al.^3^ reported that deterioration to the point of somnolence surely will become coma and recommended DSC when the level of consciousness deteriorates to somnolence. Therefore, in cases of progressive consciousness disorder, it may be desirable to intervene surgically even earlier. More recently, Kim et al.^11^ conducted a retrospective-matched case-control study on the efficacy of DSC in patients with cerebellar infarction. As a result, better clinical outcomes were obtained in patients with (1) an initial Glasgow Coma Scale score of ≥9; (2) without clinical deterioration within 72 h from the onset; (3) an infarction volume ratio between 0.25 and 0.33 according to their radiological criteria; and (4) no brainstem infarction. As they mentioned, however, DSC performed based only on the volume ratio, without deterioration of consciousness, has the risk of refusal by the patient or the family. Furthermore, their volume ratio was calculated manually using brain CT scans, which they mentioned as a limitation, in contrast to our study.

We obtained positive results in 10 of 14 (71.4%) patients, which is comparable to results in previous reports.^1–3^ Statistically significant factors influencing the prognosis included “Infartcion other than cerebellar infarction” and “ hydrocephalus” in our study (*p*<0.01 and *p*<0.05, respectively) (Table 2). Cerebral infarction was observed in the supratentorial region in three patients with a poor prognosis (cases 7, 8, 12). Brain damage in addition to the cerebellar infarction appears to be a major determinant of a poor prognosis. Persistent atrial fibrillation was observed in all these patients, and the etiology of the infarction was cardiogenic embolism. The latter prognostic factor may be relieved if DSC is undertaken as early as possible to avoid secondary brain damage by obstructive hydrocephalus, even though we found no significant difference in the time from onset to surgery.

In addition, co-morbidities (e.g., heart failure, chronic emphysema) also complicate the systemic condition, leading to a poor postoperative course (as in case 5, where only occlusion of the right posterior inferior cerebellar artery was observed).

Brainstem involvement is another issue that should be discussed. In this study, the prognosis was poor in a patient with brainstem bleeding due to hemorrhagic infarction. Some reports noted that the presence of brainstem infarction is an added cause of a poor prognosis. They noted that surgical intervention most improves the prognosis in patients without brainstem infarction.^2,3,12^

As a surgical method, there is no definitive conclusion as to whether to perform DSC, add ventricular drainage (VD) to treat the hydrocephalus, or perform only VD. Among 42 patients with cerebellar infarction, Rieke et al.^13^ evaluated 20 undergoing conservative treatments, 15 undergoing VD, and 7 undergoing DSC. Based on their study results, VD is recommended in patients with stupor due to hydrocephalus, whereas DSC should be performed in comatose cases with brainstem compression. Among 84 patients with acute cerebellar infarction with a mass effect found on head CT, Jauss et al.^14^ compared 34 patients undergoing DSC and 14 undergoing VD. They found no difference between VD and DSC. Severe sequelae, however, such as consciousness disturbance and hemiplegia, have occurred frequently when only VD is performed.^3,8,15–17^ There also is a report that the level of consciousness worsens if only VD is performed because it adds the risk of upward tentorial herniation.^18^ As already mentioned, early DSC may decrease the significance of the situation and the need for VD. Further analysis is required to clarify this dilemma with more cases in the future.

Limitations of this study include its retrospective nature and the small number of patients from a single facility. The validity of our results should be assessed in a multicenter study with more cases.

In conclusion, we described 14 patients undergoing DSC for cerebellar infarction. Our results suggest that there is a high possibility that a good prognosis could be obtained with DSC if there is no infarction other than that in the cerebellum and if complications of the whole body do not occur in the case of progressive consciousness disturbance.

## Figures and Tables

**Table1:** Characteristics of 14 patients who underwent decompressive suboccipital craniectomy for cerebellar infarction

Case	Age(years)	Sex	Onset to operation time (h:min)	Territory of infarction	Etiology	Hemorrhagic infarction	Hydrocephalus or brainstem compression	C1 laminectomy/ventricular drainage	Past history	mRS at 90 days	Infarction volume (ml^3^)
1	66	F	16:00	Lt. PICA	ATBI	N	H+B	N/N	HT, DM	2	58.4
2	65	M	21:57	Lt. PICA, Lt. AICA	U/D	N	H+B	N/N	HT, MI, CI	1	53.4
3	42	M	62:22	Bil. PICA, Bil. SCA	ATBI	N	H+B	N/Y	HU	1	87.1
4	44	M	53:00	Lt. PICA, Lt. SCA	Af	N	H+B	N/Y	Af, HT	0	40.6
5	76	M	39:27	Rt. PICA	Af	Y	B	Y/N	CPE, Af, HF	6	87.8
6	78	F	56:10	Bil. PICA	Af	Y	H+B	Y/Y	HT, DM	2	69.0
7	62	M	20:00	Rt. PICA, Rt. SCA, Lt. PCA	Af	N	H+B	N/Y	CI, Af, MI,HT	5	104.7
8	72	M	40:10	Lt. SCA, Rt. MCA	Af	Y	B	N/Y	HT, DM, Af	6	64.0
9	67	M	157:10	Lt. PICA, Bil. SCA	ATBI	Y	H	N/Y	HT	1	54.9
10	84	M	148:10	Lt. PICA	Af	Y	H+B	Y/Y	Af	2	62.8
11	59	M	38:50	Rt. PICA	Af	Y	H+B	N/Y	Af, HT	2	52.5
12	67	M	61:28	Lt. SCA, Rt. PCA	Af	Y	H+B	N/N	Af, DM, MI	4	33.4
13	64	M	31:47	Bil. PICA	ATBI	N	H+B	N/Y	CI, HT	1	74.0
14	68	M	97:20	Rt. PICA	Af	Y	H+B	N/N	Af	2	57.0

M, male; F, female; Lt, left; Rt, right; Bil, bilateral; PICA, posterior cerebellar artery; AICA, anterior inferior cerebellar artery; SCA, superior cerebellar artery; ATBI, atherothrombotic brain infarction; U/D, artery-to-artery embolic infarction by undetermined cause; Af, atrial fibrillation; N, no; Y, yes; H, hydrocephalus; B, brainstem compression; C1 laminectomy, laminectomy of the first cervical vertebra; HT, hypertension; DM, diabetes mellitus; MI, myocardial infarction; CI, cerebral infarction; HU, hyperuricemia; CPE, chronic pulmonary emphysema; HT, hypertension; mRS, modified Rankin Scale.

**Table2:** Comparison of patients with good and poor prognoses for each factor

Factor	mRS ≤2	mRS ≥3	*p*
Cases (*n*)	10	4	
Age (years)	63.7±13.1	69.3±6.1	NS**
Male sex (*n*)	8 (80%)	4 (100%)	NS**
Onset to operation time (h:min)	68:27±50	40:38±16:57	NS**
Infarction other than cerebellar infarction (*n*)	0	3	<0.01*
Cardiogenic embolism (*n*)	5	4	NS*
Hemorrhagic infarction (*n*)	5	3	NS*
Hydrocephalus (*n*)	10	2	<0.05*
History of MI (*n*)	1	2	NS*
Infarction volume (ml^3^)	61.0±13.0	72.5±31.0	NS**

MI, myocardial infarction; mRS, modified Rankin Scale; NS, not significant. * χ^2^ test. ** Student’s *t* test.

## References

[1] Feely MP. Cerebellar infarction. Neurosurgery 1979; 4: 7–11. 450221 10.1227/00006123-197901000-00003

[2] Tsitsopoulos PP, Tobieson L, Enblad P, Marklund N. Clinical outcome following surgical treatment for bilateral cerebellar infarction. Acta Neurol Scand 2011; 123: 345–351. 20636449 10.1111/j.1600-0404.2010.01404.x

[3] Ogasawara K, Koshu K, Nagamine Y, Fujiwara S, Mizoi K, Yoshimoto T. Surgical decompression for massive cerebral infarction. No Shinkei Geka 1995; 23: 43–48 (in Japanese). 7845519

[4] Jüttler E, Schwickert S, Ringleb PA, Huttner HB, Köhrmann M, Aschoff A. Long-term outcome after surgical treatment for space occupying cerebellar infarction: experience in 56 patients. Stroke 2009; 40: 3060–3066. 19574554 10.1161/STROKEAHA.109.550913

[5] Hornig CR, Rust DS, Busse O, Jauss M, Laun A. Space occupying cerebellar infarction: clinical course and prognosis. Stroke 1994; 25: 372–374. 8303748 10.1161/01.str.25.2.372

[6] Wijdicks EF, Sheth KN, Carter BS, Greer DM, Kasner SE, Kimberly WT, Schwab S, Smith EE, Tamargo RJ, Wintermark M. Recommendations for the management of cerebral and cerebellar infarction with swelling: a statement for healthcare professional from the American Heart Association/American Stroke Association. Stroke 2014; 45: 1222–1238. 24481970 10.1161/01.str.0000441965.15164.d6

[7] The Japan Stroke Society. Japanese Guideline for Management of Stroke 2015. Tokyo: Kyowa-Kikaku; 2015: 66 (in Japanese).

[8] Khan M, Polyzoidis KS, Adegbite AB, McQueen JD. Massive cerebellar infarction: “conservative” management. Stroke 1983; 14: 745–751. 6658959 10.1161/01.str.14.5.745

[9] Jüttler E, Schwab S, Schmiedek P, Unterberg A, Hennerici M, Woitzik J, Witte S, Jenetzky E, Hacke W. Decompressive surgery for the treatment of malignant infarction of the middle cerebral artery (DESTINY): a randomized, controlled trial. Stroke 2007; 38: 2518–2525. 17690310 10.1161/STROKEAHA.107.485649

[10] Tsitsopoulos PP, Tobieson L, Enblad P, Marklund N. Clinical outcome following surgical treatment for bilateral cerebellar infarction. Acta Neurol Scand 2011; 123: 345–351. 20636449 10.1111/j.1600-0404.2010.01404.x

[11] Kim MJ, Park SK, Song J, Oh SY, Lim YC, Sim SY, Shin YS, Chung J. Preventive suboccipital decompressive craniectomy for cerebellar infarction: a retrospective-matched case-control study. Stroke 2016; 47: 2565–2573. 27608818 10.1161/STROKEAHA.116.014078

[12] Tchopev Z, Hiller M, Zhuo J, Betz J, Gullapalli R, Sheth KN. Prediction of poor outcome in cerebellar infarction by diffusion MRI. Neurocrit Care 2013; 19: 276–282. 23921570 10.1007/s12028-013-9886-2

[13] Rieke K, Krieger D, Adams HP, Aschoff A, Meyding-Lamadé U, Hacke W. Therapeutic strategies in space-occupying cerebellar infarction based on clinical neuroradiological and neurophysiological data. Cerebrovasc Dis 1993; 3: 45–55.

[14] Jauss M, Krieger D, Hornig C, Schramm J, Busse O. Surgical and medical management of patients with massive cerebellar infarctions: results of the German-Austrian Cerebellar Infarction Study. J Neurol 1999; 246: 257–264. 10367693 10.1007/s004150050344

[15] Andoh T, Sakai N, Yamada H, Hattori T, Miwa Y, Hirata T, Tanabe Y, Ohkuma A, Funakoshi T, Takada M. Cerebellar infarction: analysis of 33 cases. No Shinkei Geka 1990; 18: 821–828 (in Japanese). 2234303

[16] Auer LM, Auer T, Sayama I. Indication for surgical treatment of cerebellar haemorrhage and infarction. Acta Neurochir (Wien) 1986; 79: 74–79. 3962746 10.1007/BF01407448

[17] Laun A, Busse O, Calatayud V, Klug N. Cerebellar infarcts in the area of the supply of the PICA and their surgical treatment. Acta Neurochir (Wien) 1984; 71: 295–306. 6741640 10.1007/BF01401324

[18] Hara Y, Hosoda K, Ohta K. Upward transtentorial herniation associated with severe posterior fossa subarachnoid hemorrhage due to vertebral artery dissecting aneurysm: a report of two cases. Japanese Journal of Neurosurgery (Tokyo) 2007; 16: 423–427 (in Japanese).

